# A Case Report of Successful Treatment of Minoxidil Toxicosis Using Hemodialysis in a Cat

**DOI:** 10.3390/vetsci11100487

**Published:** 2024-10-09

**Authors:** Woonchan Ahn, Taeho Lee, Soyoung Jung, Aryung Nam

**Affiliations:** 1 Department of Veterinary Internal Medicine, College of Veterinary Medicine, Kangwon National University, Chuncheon 24341, Republic of Korea; ahnwoonchan@smartah.co.kr; 2 Smart Animal Hospital Sinsa, Seoul 06026, Republic of Korea; leetaeho@smartah.co.kr (T.L.); jeung1014@konkuk.ac.kr (S.J.); 3 College of Veterinary Medicine, Chungnam National University, Daejeon 34134, Republic of Korea; 4 Department of Veterinary Internal Medicine, College of Veterinary Medicine, Konkuk University, Seoul 05029, Republic of Korea

**Keywords:** case report, cat, hemodialysis, minoxidil, toxicosis

## Abstract

**Simple Summary:**

Minoxidil is an FDA-approved drug used for human hair regrowth. Minoxidil toxicosis can occur and be fatal in dogs and cats and presents with symptoms such as lethargy, anorexia, vomiting, hypotension, and tachypnea. We described a case of minoxidil toxicosis in a cat after accidental exposure to topical minoxidil presenting with severe clinical signs, including respiratory distress and hemodynamic instability. Chest X-rays showed mild lung infiltration and pleural effusion. Initial treatments with oxygen, fluids, furosemide, and dopamine were ineffective. However, hemodialysis proved effective in improving the patient’s condition and facilitating a full recovery. This case is the first to document successful hemodialysis treatment for minoxidil toxicosis in a cat, thus highlighting the potential therapeutic role of hemodialysis in minoxidil toxicosis.

**Abstract:**

A 5-year-old castrated male American Shorthair cat presented with lethargy and anorexia after accidentally knocking over a bottle of topical minoxidil and spilling it onto its body. Physical examination revealed rapid shallow breathing, pale mucous membranes, hypothermia, tachycardia, and hypotension. Thoracic radiography revealed mild pulmonary infiltration and pleural effusion. Despite conservative treatment, including oxygen therapy, and intravenous fluid, furosemide, and dopamine administration, the patient showed no improvement. After two sessions of intermittent hemodialysis, the cat’s respiratory pattern and overall condition gradually improved; normal body temperature and blood pressure were achieved. The cat recovered fully and was discharged on the 11th day of hospitalization. This is the first report on the use of hemodialysis in the treatment of a cat with minoxidil toxicosis.

## 1. Introduction

Minoxidil is a Food and Drug Administration (FDA)-approved drug that is widely used in human medicine to promote hair regrowth and prevent hair loss. Initially prescribed as an oral medication for refractory systemic hypertension, topical minoxidil may be systemically absorbed, leading to adverse effects, including hypotension, tachycardia, myocardial infarction, elevated liver enzymes, and fetal malformations [1,2,3,4]. Minoxidil toxicosis may occur in dogs and cats, commonly presenting with symptoms such as lethargy, anorexia, vomiting, hypotension, and tachypnea, which can be fatal [5,6,7].

Hemodialysis, an extracorporeal therapy, is used to treat acute poisoning or drug overdoses. During this procedure, a substance or its metabolites are eliminated from the blood mainly via diffusion across a semipermeable membrane, facilitated by a concentration gradient [8]. Hemodialysis has been used successfully in the management of intoxication associated with ethylene glycol, baclofen, phenobarbital, and amikacin in dogs and cats [9,10,11]. Herein, we report the successful recovery of a cat with minoxidil toxicosis after hemodialysis, thus highlighting this treatment modality as a potential treatment option in veterinary medicine.

## 2. Case Description

A 5-year-old castrated male American Shorthair cat weighing 5 kg was exposed to its owner’s topical minoxidil when it accidentally knocked over the solution bottle, thereby spilling it onto its body and licking it. The cat subsequently presented with acute lethargy and anorexia and showed no signs of urination. Consequently, the cat presented to the SMART animal hospital Sinsa 24 h after the minoxidil exposure. On physical examination, it exhibited rapid and shallow breathing (respiratory rate: 60 breaths/min) and pale mucous membranes. The cat also presented with signs of hypothermia (rectal temperature: 36.3 °C), along with mild tachycardia (204 beats/min; reference range: 100–140 beats/min) and hypotension (systolic blood pressure: 80 mmHg; reference range: 90–140 mmHg; Doppler method).

**Figure 1:**
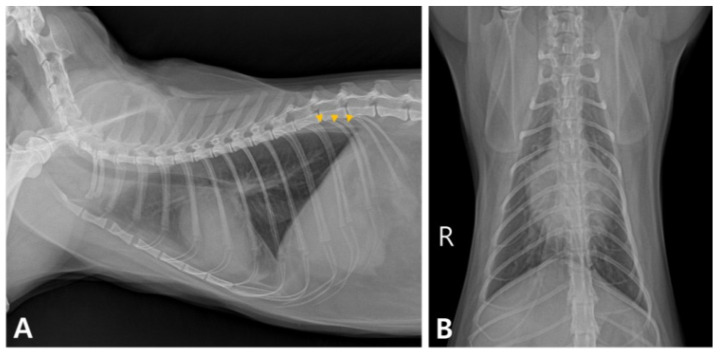
Thoracic radiographs on admission. (**A**) The caudal lung lobes were retracted from the thoracic wall (arrowheads). (**B**) Mild bronchointerstitial pulmonary patterns were observed.

The complete blood count was within the reference interval. Serum biochemistry revealed an elevated blood urea nitrogen level of 44 mg/dL (reference range: 16–36 mg/dL), hyponatremia (146 mmol/L; reference range: 149–157 mmol/L), and hypochloremia (106 mmol/L; reference range: 117–127 mmol/L). Thoracic radiography revealed a mild bronchointerstitial pulmonary pattern and retraction of the caudal lung lobes from the thoracic wall (Figure 1). Based on the cat’s history and previous annual check-up results, including normal pro-BNP levels and no abnormalities in chest radiographs, a provisional diagnosis of minoxidil toxicosis was made.

**Figure 2:**
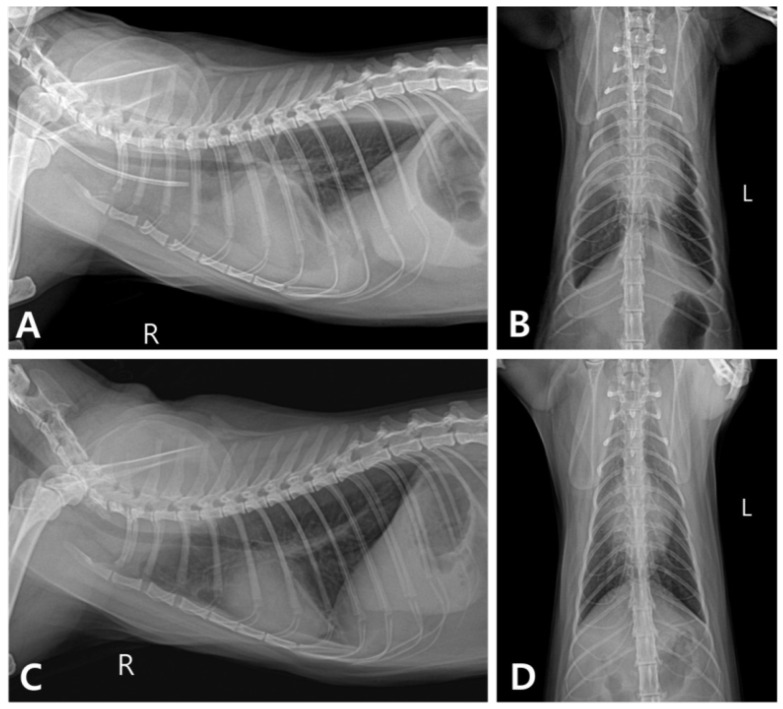
Thoracic radiographs taken after the second intermittent hemodialysis and before discharge. (**A**,**B**) Bilateral pleural effusions were noted with the right side being more severe. (**C**,**D**) The pleural effusions had resolved by the time of discharge.

**Figure 3:**
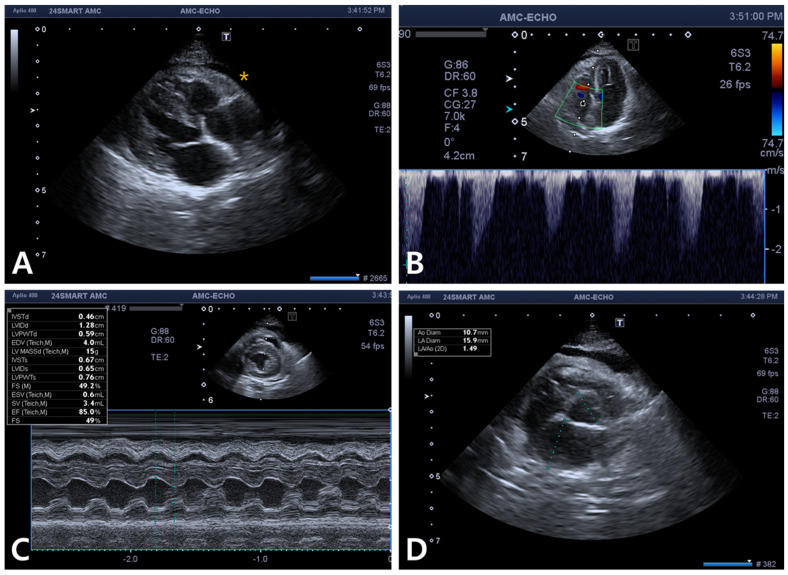
Echocardiogram taken after the second intermittent hemodialysis. (**A**) Right ventricular dilatation and a small amount of pericardial effusion (asterisk) in the right parasternal long-axis view. (**B**) Tricuspid regurgitation approximates 2.3 m/s (in the left apical 4-chamber view). (**C**,**D**) No abnormalities were observed in the left atrium and left ventricle.

The cat was hospitalized in the intensive care unit with heated pads used to help restore the cat’s normal body temperature and received oxygen therapy and intravenous fluids. Furosemide (1 mg/kg; Lasix, Handok Inc., Seoul, Republic of Korea) was intravenously administered to alleviate pleural effusion. A continuous rate infusion (CRI) of dopamine (Huons Dopamine Inj., Huons, Seongnam, Republic of Korea) was administered at a rate of 5 μg/kg/min to improve hypotension and increased to 10 μg/kg/min. Despite treatment, the cat’s clinical symptoms did not improve, and its blood pressure decreased to 70 mmHg. Hemodialysis was initiated on the second day of hospitalization with the owner’s consent. Intermittent hemodialysis (IHD) sessions were performed using a Fresenius multifiltrate system (Fresenius Medical Care AG & Co. KGaA, Bad Homburg, Germany) using polysulfone dialyzers (Ultraflux^®^ AV paed, Fresenius Medical Care AG & Co. KGaA, Bad Homburg, Germany) and the pediatric continuous venovenous hemodialysis (CVVHD) mode. Under sedation with propofol (Provive Inj., Pharmbio Korea Inc., Seoul, Republic of Korea) at CRI of 0.2 mg/kg/min, vascular access via the right jugular vein was obtained using an 8 Fr-sized 2-lumen central venous catheter set (Blue FlexTip^®^ ARROWgard^®^ Blue catheter, Arrow international, Inc., Cleveland, OH, USA). Butorphanol (Butorphan Injection, Myungmoon Pharm, Seoul, Republic of Korea) was prescribed for analgesia as needed. The average blood flow rate was 25 mL/min, and the dialysate flow rate was maintained at 500 mL/h during each 4 or 5 h session. The amounts of blood processed were 1.2 and 1.5 L/kg in the 4 and 5 h sessions, respectively. MultiBic^®^ dialysate (Fresenius Medical Care Deutschland GmbH, Wendel, Germany) was used, and ultrafiltration was carefully performed to reduce the patient’s fluid retention caused by intoxication. The target dry weight was 4.7 kg, and the ultrafiltration rate was initially 5 mL/h and gradually increased to 25 mL/h. To prevent unstable hemodynamic changes, such as decreasing blood pressure, 40 mL of type A feline blood was primed for an extracorporeal circuit before each session. After two sessions of IHD over 2 consecutive days, the cat showed a slight improvement in overall condition and appetite. As the body temperature and blood pressure returned to the normal range, the dopamine CRI was tapered and discontinued (Supplementary Figure S1). Pleural effusion was still evident on chest radiography (Figure 2A,B), and echocardiography revealed right ventricular dilatation, tricuspid regurgitation, and a small amount of pericardial effusion (Figure 3). To resolve these issues, the cat’s treatment plan was adjusted to include furosemide CRI at a rate of 0.5 mg/kg/h and oral pimobendan (1.25 mg/cat q12h; Vetmedin, Boehringer Ingelheim, Ingelheim, Germany) and spironolactone (1 mg/kg q12h; Spiracton Tab, Daewon Pharmaceutical, Seoul, Republic of Korea). The cat’s respiratory pattern gradually improved, and follow-up radiography demonstrated a reduction in pleural effusion (Figure 2C,D). The furosemide CRI was gradually reduced and changed to oral medication 5 days after the second IHD session. The cat eventually recovered fully and was discharged on the 11th day of hospitalization. Follow-up blood tests and thoracic radiography conducted 7 days after discharge showed no abnormal findings.

## 3. Discussion

Minoxidil was initially prescribed for the management of resistant or severe hypertension in human patients [1,12]. The drug binds to adenosine triphosphate-sensitive potassium channels in the arteriolar vascular smooth muscle cells. The activation of these channels leads to potassium efflux, resulting in the relaxation of smooth muscle cells and subsequent vasodilation [12,13]. Minoxidil causes sodium and water retention; therefore, it is commonly administered in combination with loop diuretics for hypertension [12]. In addition, beta-blockers may be prescribed alongside minoxidil to prevent reflex tachycardia [12]. However, because it has the promotion of hair growth as a side effect, minoxidil has been widely used to treat pattern hair loss, often as a topical formulation [13,14].

A small amount of topical minoxidil can result in clinical symptoms associated with cardiovascular injury in dogs and cats, which tend to be more severe and have a higher mortality rate in cats than in dogs [5,7]. However, only a few such cases have been documented in cats. Among them, two led to death, one recovered with supportive treatment, and two cats from the same household were treated with vasopressors and intravenous lipid emulsions [5,15,16]. These reports emphasize the importance of closely monitoring the cardiovascular and respiratory systems, providing oxygen supplementation, administering diuretics for pulmonary edema and vasopressors for hypotension, and performing thoracocentesis if necessary for pleural effusion. Intravenous fluids are recommended to promote urine production and aid in the elimination of drugs and their metabolites [5]. In our case, furosemide and spironolactone, along with pimobendan, were used to manage the effusion and address myocardial damage caused by minoxidil intoxication. Although pimobendan, a positive inotropic drug, is not approved for use in cats and has controversial clinical benefits, it is known to be well tolerated in cats with cardiomyopathy and congestive heart failure [17,18,19].

Decontamination, intravenous lipid emulsion therapy, and hemodialysis are considered to resolve the toxicosis based on the exposure route and duration of exposure to the toxic substance. Previous reports have described the successful use of hemodialysis to treat poisoning caused by substances such as ethylene glycol, phenobarbital, amikacin, and baclofen in cats [9,10,11]. Minoxidil is primarily metabolized in the liver to minoxidil-O-glucuronide and minoxidil-N-O-sulfate, which are excreted in the urine through glomerular filtration [20,21]. For a substance to be effectively eliminated through hemodialysis, it should have certain characteristics, including a low molecular weight, high solubility in water, low protein binding, and low volume of distribution [22]. Minoxidil has a molecular weight of 209.25 Daltons and water solubility of 2.2 mg/mL [20,23]. The drug is distributed throughout the body, with a volume distribution of approximately 2.8–3.3 L/kg [21]. However, minoxidil is minimally bound to proteins, indicating that it is freely available for removal by dialysis. Consequently, hemodialysis can effectively remove minoxidil and its metabolites. In addition, ultrafiltration can reduce the fluid retention caused by minoxidil, thereby reducing the required diuretic dosage. For these reasons, the cat received hemodialysis. In addition, among the modalities for hemodialysis, we selected IHD, which is designed for relatively short and discontinuous (4–5 h) yet intense treatments and is considered to have superior efficiency for toxin removal compared to continuous renal replacement therapy [24]. After two sessions of hemodialysis, the patient’s vital signs improved; however, additional hospitalization was required to treat the persistent pleural effusion. The amount of exposure was unknown and was only described in terms of licks, as in previous cases of minoxidil toxicosis [7]. The dose per body weight has only been calculated in 10 dogs, with the lowest dose at which clinical signs developed being 0.79 mg/kg. Even a very small amount of topical minoxidil (fewer than a few drops) can lead to severe clinical symptoms and death in cats [5,7]. Because the cat in the present report had a substantial amount of minoxidil spilled from a broken bottle onto its body and licked it, it was presumed to have been exposed to a much larger amount of minoxidil than in previously reported cases. Therefore, the prognosis would have been worse if hemodialysis had not been performed.

In this instance, the blood minoxidil concentration was not measured because a commercial laboratory was not available. Therefore, the inability to verify the efficacy of hemodialysis through a reduction in blood minoxidil levels is a limitation of this case report. In several cases of intoxication treatment using hemodialysis, the concentration of the toxic substance is measured before, during, and after hemodialysis to assess its removal [9,10,11]. Amikacin clearance was calculated from serial blood samples collected during hemodialysis from a cat that was mistakenly administered 400 mg of amikacin [11]. The serum amikacin concentration at the initiation of hemodialysis was three times higher than the concentration predicted based on pharmacokinetics in cats. This may be due to changes in pharmacokinetics or a significant decrease in the GFR resulting from an overdose. In a cat with phenobarbital overdose, the phenobarbital concentration gradually decreases during hemodialysis [10]. Johnson et al. recently measured blood minoxidil concentrations in two cats with minoxidil toxicosis before and after lipid emulsion administration [15]. They suggested that it is impossible to determine a normal rate of excretion because minoxidil blood concentrations have not yet been reported in cats, and a discrepancy might arise between serum and plasma minoxidil concentrations from sample handling issues or the pharmacokinetics/pharmacodynamics of minoxidil in cats.

## 4. Conclusions

To our knowledge, this is the first report describing the successful treatment of a cat exposed to topical minoxidil using hemodialysis. Prompt hemodialysis can lead to a more favorable prognosis for cats with minoxidil toxicosis.

## Data Availability

The data that support the findings of this study are available from the corresponding author upon reasonable request.

## Supplementary Materials

The following supporting information can be downloaded at: https://www.mdpi.com/article/10.3390/vetsci11100487/s1, Supplementary Figure S1. Clinical timeline of the cat. After two sessions of hemodialysis (HD, indicated by arrows), the cat’s systolic blood pressure and body temperature returned to the normal range. The dopamine continuous rate infusion (CRI) was tapered and subsequently discontinued as shown in the flow.
