# Supplementary figures and images for: A Case Report of Successful Treatment of Minoxidil Toxicosis Using Hemodialysis in a Cat
Source: Vet Sci. 2024 Oct 9;11(10):487. doi: 10.3390/vetsci11100487 (PMC11511512; doi:10.3390/vetsci11100487)

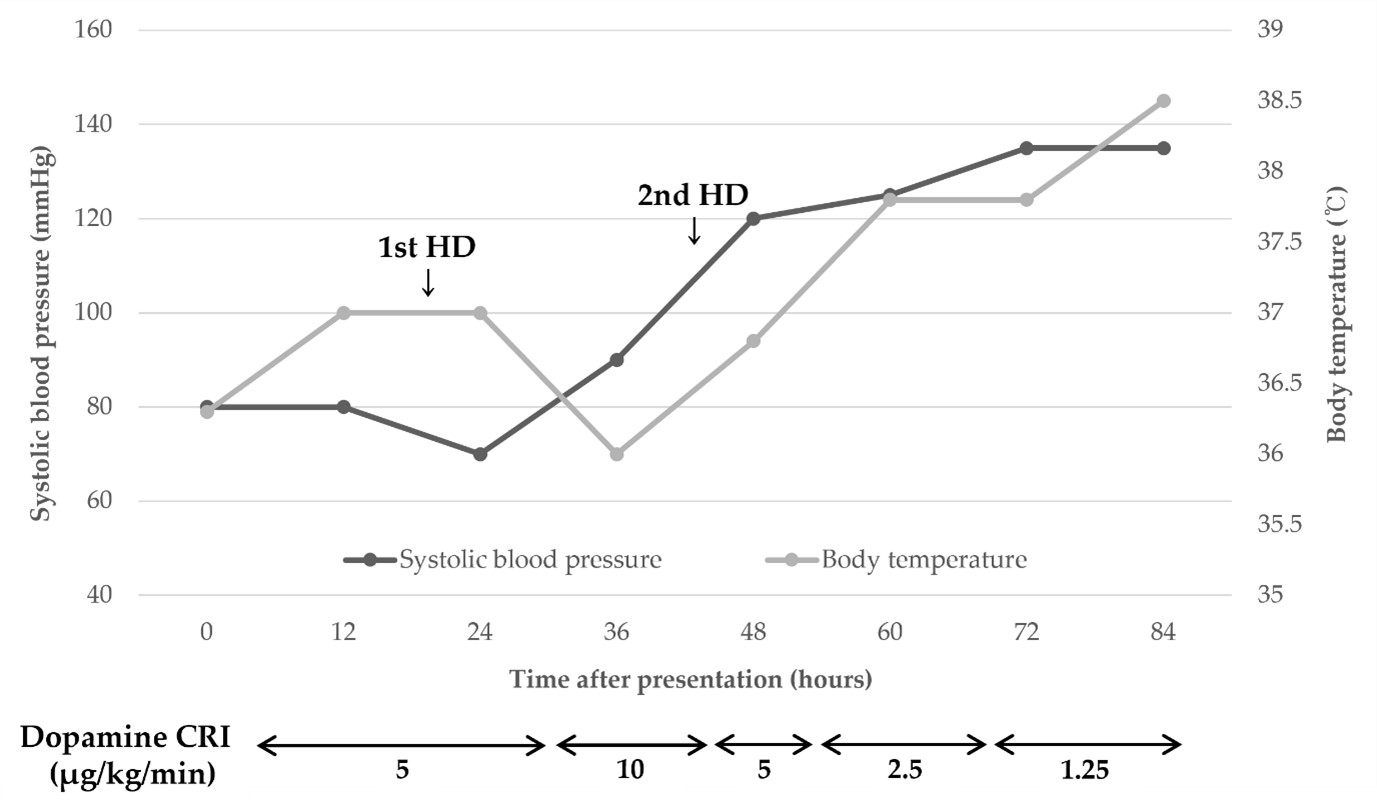

Supplement: Supplementary file 1 [file vetsci-11-00487-s001.zip › vetsci-3137630-supplementary.jpg]
